# Optimization of an amplicon sequencing‐based microsatellite panel and protocol for stock identification and kinship inference of lake trout (*Salvelinus namaycush*)

**DOI:** 10.1002/ece3.10020

**Published:** 2023-04-18

**Authors:** Benjamin Marcy‐Quay, Chris C. Wilson, Christopher A. Osborne, J. Ellen Marsden

**Affiliations:** ^1^ Rubenstein Ecosystem Science Laboratory University of Vermont Burlington Vermont USA; ^2^ Ontario Ministry of Natural Resources and Forestry Trent University Peterborough Ontario Canada; ^3^ Department of Biological Sciences University at Buffalo Buffalo New York USA

**Keywords:** half‐sibling, lake charr, mixed‐stock assignment, parent‐offspring, population genetics

## Abstract

Kinship‐based methods of population assessment such as close‐kin mark‐recapture require accurate and efficient genotyping methods capable of resolving complex relationships among kin. Inference of such relationships can be difficult using biallelic loci due to the large number of markers required to obtain the necessary power. Sequencing‐based microsatellite panels offer an efficient alternative, combining high polymorphism with efficient next‐generation methods. Here we construct, optimize, and test one such panel for lake trout (*Salvelinus namaycush*) using a combination of previously‐published loci adapted for sequencing and de novo loci mined from a genome assembly. We performed three rounds of primer optimization, yielding a final panel of 131 loci, followed by testing with two different levels of PCR multiplexing (all primers in one or two groups) and two different reaction volumes (5 and 10 μL). Our results showed that the use of the largest multiplex and smallest reaction volume did not substantially change results, allowing significant cost and time savings. To test panel accuracy, we used both a set of 153 known‐origin samples from origins of management interest and a series of hatchery crosses representing nine families with parent‐offspring, half‐sibling, and largely‐unrelated pairs. Our results indicate that sequencing‐based microsatellite panels can efficiently and accurately provide the information required for a population genetics analyses including population assignment, calculation of between‐population *F*_ST_, and kinship‐based population estimation techniques. Such techniques are seeing increasing applications for a wide range of taxa; our findings should provide insight and guidance for the development of the necessary molecular resources.

## Graphical abstract

Kinship‐based methods of population assessment such as close‐kin mark‐recapture require accurate and efficient genotyping methods capable of resolving complex relationships among kin, but inference of such relationships can be difficult using biallelic loci. Sequencing‐based microsatellite panels offer an efficient alternative, combining high polymorphism with efficient next‐generation methods. Here we construct, optimize, and test one such panel for lake trout (*Salvelinus namaycush*) using a combination of historical loci adapted for sequencing and de novo loci mined from a genome assembly.

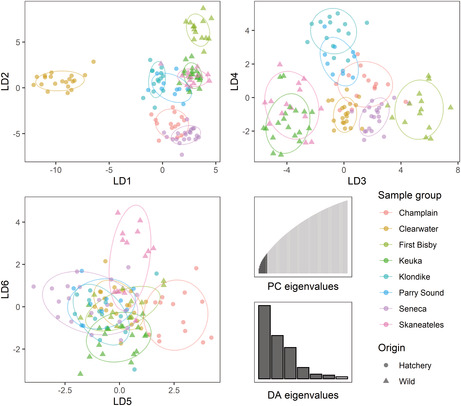

## 1 INTRODUCTION

Genetic markers have been used for several decades to assess stock composition, survival, and recruitment in lake trout (*Salvelinus namaycush*), a long‐lived piscivore native to Canada and parts of the northern United States (Goetz et al., 2010; Grewe et al., 1993; Krueger et al., 1989; McDermid et al., 2020; Page et al., 2003, 2004). A major focus of these efforts has been population management in the Laurentian Great Lakes (hereafter, Great Lakes), where a combination of intensive commercial fishing, spawning habitat degradation, and invasion by sea lamprey (*Petromyzon marinus*) eradicated or severely reduced the native stocks by the 1950s (Christie, 1973; Coble et al., 1990; Cornelius et al., 1995). Efforts to restore lake trout populations began in the 1960s with stocking of multiple strains to maximize genetic diversity and included evaluation of strain success with the creation of an allozyme panel capable of differentiating among strains stocked in Lake Ontario (Krueger et al., 1989; Marsden et al., 1989) and similar work in the upper Great Lakes following the development of microsatellite markers (Page et al., 2003, 2004). Genetic analyses have also revealed the negative effects of hatchery stocking on overall genetic diversity in lake trout within both the Great Lakes (Guinand et al., 2003) and smaller waters across their native range (Valiquette et al., 2014). Negative effects of lake trout invasions on native species and ecosystems in many western lakes in the United States (Crossman, 1995; Martinez et al., 2009) have additionally prompted the development of marker panels aimed at estimating the source and size of founding populations (Kalinowski et al., 2010; Rollins et al., 2009).

Due to the rapid evolution of molecular biology during the period encompassing the aforementioned research and the wide range of agencies and institutions involved, it is not surprising that there has been little standardization of marker panels among researchers. Standardization of microsatellite scoring among labs has historically posed multiple challenges which take significant effort to overcome (Ellis et al., 2011; Moran et al., 2006; Seeb et al., 2007; Welsh & May, 2006). Studies using electrophoresis‐based microsatellites have adapted markers from other species (Guinand et al., 2003; Northrup et al., 2010; Page et al., 2003), developed new markers (Rollins et al., 2009), or used some combination of the two (Baillie et al., 2016; Larson et al., 2020; Markham et al., 2022; McCracken et al., 2013; McDermid et al., 2020; Scribner et al., 2018). With the advent of next‐generation sequencing, studies have also employed restriction site‐associated DNA sequencing (RADseq), again with widely differing sets of loci (Bernatchez et al., 2016; Euclide et al., 2022; Morissette et al., 2019).

Recent observations of wild lake trout reproduction and recruitment in Lake Michigan (Hanson et al., 2013), Lake Huron (Johnson et al., 2015), Lake Champlain (Marsden et al., 2018), Lake Ontario (Gatch et al., 2021), and Lake Erie (Markham et al., 2022) have raised new questions about the parental source of recruits, leading to another round of genetic stock identification studies (e.g., Larson et al., 2020; Scribner et al., 2018). In addition, new techniques such as parentage‐based tagging (PBT) and close‐kin mark‐recapture (CKMR) have expanded the utility of genetic studies for lake trout conservation and management. In particular, PBT provides a more economical method for studying the relative performance of stocked fish by matching captured individuals to their hatchery‐broodstock parents (Steele et al., 2019) while CKMR allows the estimation of absolute abundance and survival of wild parental populations based on the prevalence of parent‐offspring and half‐sibling pairs among captured fish (Bravington, Skaug, & Anderson, 2016; Marcy‐Quay et al., 2020). Both techniques use the same base information, identification of kin pairs through genotyping, but target different portions of a population. PBT's main utility is strongly linked to hatchery production (where all parents are known and can be sampled), whereas CKMR is most useful for wild recruitment where the parental dynamics are unknown and of interest.

Kinship‐based approaches like CKMR and PBT, however, require much more powerful marker panels than previous methods to resolve subtle differences between closely‐related individuals (Hauser et al., 2011). Panels based on high numbers of single‐nucleotide polymorphisms (SNPs) are generally more powerful than historical microsatellite panels that use relatively few markers, but suffer from their own disadvantages including high startup costs for the purchase of reagents. For example, both amplicon‐based techniques such as Genotyping‐in‐Thousands by sequencing (GTseq) (Campbell et al., 2015) and sequence capture approach such as RAD Capture (Rapture) (Ali et al., 2015) require the purchase of several hundred primer pairs or capture baits (Meek & Larson, 2019). Less targeted methods such as restriction‐site associated DNA sequencing (RADseq) (Baird et al., 2008) avoid these reagent costs but, as a consequence, incur high per‐sample costs for sequencing (Meek & Larson, 2019). Of these options, GTseq is perhaps the most economical option for genotyping of mid‐sized datasets (several hundred samples) but the typically‐biallelic nature of SNP markers means that a high number of loci are needed to resolve complex relationships. This, in turn, risks reductions in inferential power due to physical linkage among a large number of loci used and ultimately hinders the ability of SNPs panels to resolve half‐sibling relationships unless combined into multi‐allelic microhaplotypes (Baetscher et al., 2018). More recently, sequencing‐based microsatellite panels have emerged as an economical alternative as their inherently higher polymorphism allows for reduced reagent and sequencing costs while still providing high inferential power capable of resolving complex relationships and facilitating standardization among multiple labs (Layton et al., 2020). Both SNPs and sequencing‐based microsatellites have been used in previous studies that implemented CKMR (e.g., Hillary et al., 2018; Prystupa et al., 2021) and PBT (e.g., Fitzpatrick et al., 2023; Steele et al., 2019). However, while PBT is essentially focused on parent‐offspring pairs (although see Delomas & Campbell, 2022), CKMR can make use of half‐sibling pairs and studies seeking do so using GTseq typically employ panels with several thousand loci. While a GTseq panel for lake trout has been developed (Smith, 2021), it comprises only 300 loci and is designed to resolve inter‐ rather than intra‐population differences.

To address this gap, we developed a sequencing‐based microsatellite panel for lake trout capable of resolving both population‐ and kin‐level relationships. Our primary objective was to create a cost‐effective panel that could be applied to answer research questions across the species' present‐day range using a variety of techniques including CKMR and PBT. We, therefore, (1) tested two modifications to the sample preparation protocol aimed at reducing reagent usage and preparation time, (2) validated the population discrimination ability of the resulting panel using eight known‐source populations including the historically difficult‐to‐separate Champlain and Seneca strains, and (3) validated the panel's kinship inference ability using a set of hatchery crosses produced from feral Lake Champlain adults.

## 2 METHODS

### 2.1 Samples

**FIGURE 1:**
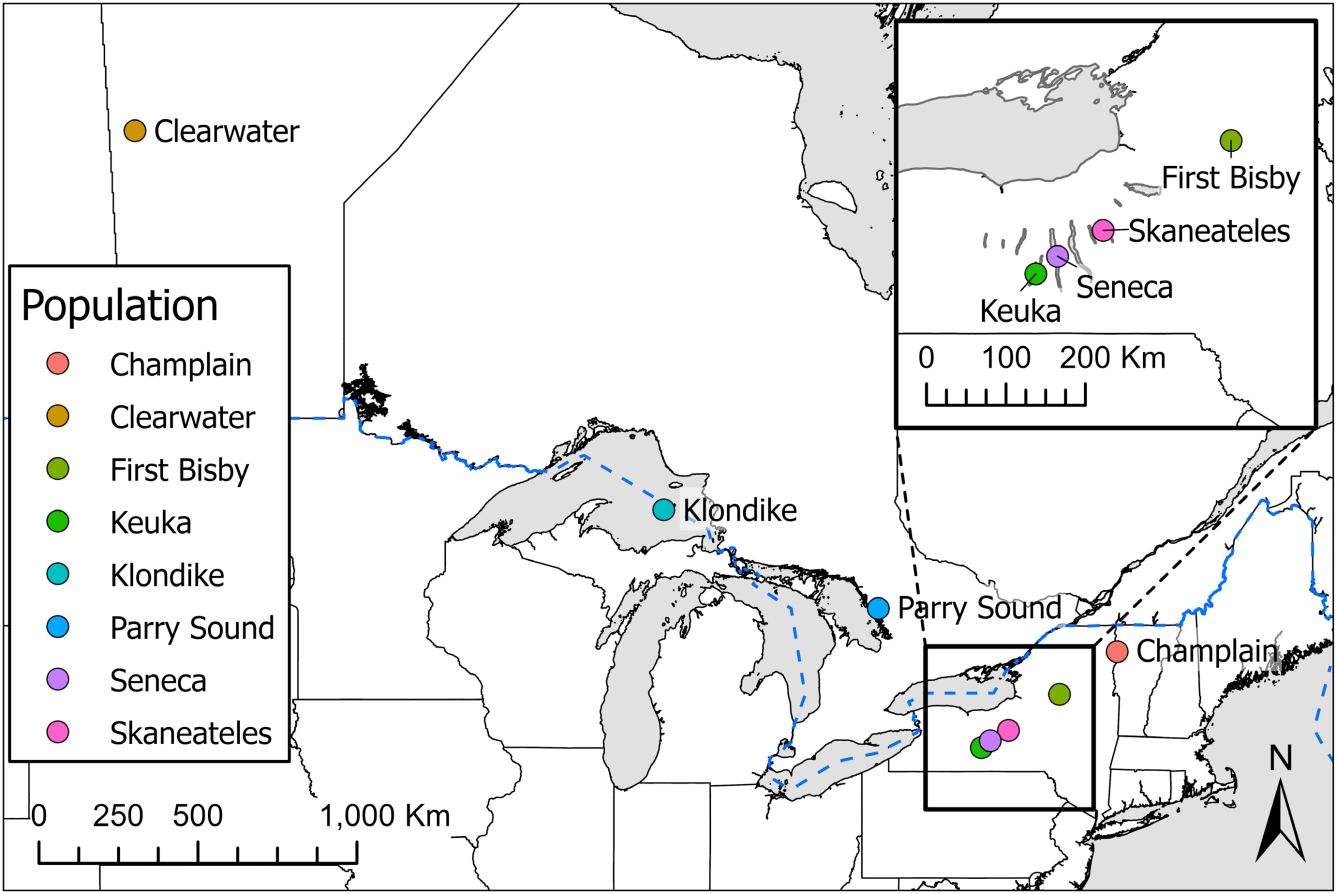
Source locations for populations included in the known‐origin sample set. Dashed blue line represents the international border between Canada and the United States.

We chose known‐origin validation samples from a range of lake trout hatchery strains and wild populations including many relevant to contemporary management decision‐making (Figure 1). These samples included the Klondike strain, a hatchery strain of the deepwater ecotype originating from Lake Superior that was previously stocked in Lake Erie (Rogers et al., 2019), and is currently stocked in Lake Ontario (NYSDEC Bureau of Fisheries, 2020); the Parry Sound strain, a hatchery isolate of a remnant native population from Lake Huron (Muir et al., 2013); the Clearwater hatchery strain, originating from a native population from Clearwater Lake, Manitoba, stocked throughout the Great Lakes during early restoration efforts (Grewe et al., 1993); and the Seneca hatchery strain, originally from the Finger Lakes of New York and now widely stocked throughout the Great Lakes (Muir et al., 2013). In addition, we included samples from natural origin populations in two other lakes in the Finger Lakes region, Keuka and Skaneateles, and from an isolated population in New York's Adirondack Mountains, First Bisby Lake. The latter is thought to be a native population (Thill, 2014), although there exists the possibility of introgression from Lake Huron‐origin fish stocked in the late 1800s (Mather, 1886). Samples were fin clips from hatchery stock (Champlain, Clearwater, and Seneca strains), and either fin clips from wild‐caught fish (First Bisby Lake), or muscle plugs from wild‐caught fish (Klondike, Parry Sound, Keuka Lake, and Skaneateles Lake individuals). Fin clips were preserved in 95% ethanol until DNA extraction while muscle plugs were frozen at −80°C immediately after sampling before being transferred to 95% ethanol approximately 1 month prior to extraction. Sample collection years ranged from 2016 to 2022 depending on the strain involved, with Keuka and First Bisby lakes representing the oldest samples (2016 and 2017, respectively) and Clearwater the most recent (2022). Each known‐origin sample set was comprised of 20 individuals with the exception of Parry Sound for which only 13 samples were available.

To create the kinship validation dataset, eggs, and milt were collected from six wild‐caught Lake Champlain fish (three males and three females) captured in trap nets at Gordon Landing (Grand Isle, VT, USA) during annual spawning surveys conducted by the Vermont Fish and Wildlife Department. Collected gametes were used to create nine families representing all possible combinations of the six individuals. A total of 216 fertilized eggs (24 per family) were individually distributed into 24‐well cell culture microplates filled with sterile reconstituted freshwater medium (ISO 6341, 2012) and incubated at 7.0°C in climate‐controlled chambers (Memmert IPP260Plus) until hatching, a standard approach that has been used to incubate similar coldwater species (Mari et al., 2016; Stewart et al., 2021). Following hatching, larvae were euthanized and preserved in 95% ethanol for later analysis. From this set, three individuals from each family were chosen for genotyping for a total of 27 known‐origin samples plus the six‐sample parental set. Larval incubation and euthanasia were conducted under UVM IACUC protocol number 202100062.

### 2.2 Panel development

We created a candidate set of 200 microsatellite loci with a target amplicon size of 180–200 bp to facilitate genotyping using 150 bp paired‐end (PE150) sequencing (Table S1). The target number of 200 was chosen based on previous experience with panels targeting 100 microsatellite loci, which provided ample power for the identification of parent‐offspring pairs but not half‐siblings. No additional attempts were made to maximize FST or polymorphism within or between any sample groups in order to provide the most unbiased information on differentiation possible. The set included 175 de novo loci generated from a lake trout reference genome (Smith et al., 2022) using Krait (Du et al., 2018) to identify and design primers for di‐, tri‐, and tetra‐repeats within the desired size range. Loci were selected from those meeting the desired characteristics using a stratified‐random design to allocate loci evenly between chromosomes while seeking to minimize the number of loci located in close proximity on the same chromosome to avoid issues with physical linkage. A further 25 loci used in previous studies and with a maximum reported length < 225 bp were included to facilitate comparison with other datasets.

The candidate model set was then refined to eliminate overamplifying loci using three successive runs of sequencing following a similar strategy to that employed by Bootsma et al. (2020) for the development of their walleye (*Sander vitreus*) GTseq panel. Conditions for these sequencing runs were as described in the “genotyping” section below with the exceptions that all runs used (1) the same sample set of 40 Champlain‐ and Seneca‐strain individuals and (2) 10 μL PCR1 reaction volumes conducted with two primer multiplexes. Sequencing was performed using an Illumina MiniSeq platform and a 300‐cycle Mid Output kit yielding approximately 2.4 Gb of output per run for an idealized coverage of 1000× per sample‐locus combination. A total of 16 loci were removed for overamplification and a further 53 failed to reliably amplify, yielding a final set of 131 loci. The final set contained 13 previously‐published loci and 118 de novo loci, representing candidate success rates of 52% and 66%, respectively.

### 2.3 Genotyping

We extracted DNA from all 186 samples using a modified version of the HotSHOT technique (Truett et al., 2000). Briefly, we cut a 1 × 2 mm section of tissue from each sample using a sterile technique and placed it into a 96‐well microplate. To this, we added 50 μL of alkaline lysis reagent (50 mM NaOH, pH 12.0) and then incubated the plate at 95°C for 30 minutes using a thermal cycler. We then cooled the plate to 4.0°C and added a 50 μL aliquot of neutralization reagent (40 mM Tris–HCl, pH 4.0) to each well. The resulting solution (extracted DNA in buffer) was stored at −20°C.

We performed polymerase chain reaction (PCR) amplification of target loci using a two‐step procedure in which an initial reaction was performed to amplify the target sequences (PCR1) followed by a second step to add index adapter sequences (PCR2). To test potential optimizations to the protocol, we created four uniquely‐indexed post‐PCR samples for each DNA sample representing all combinations of two PCR1 reaction volumes (5 μL and 10 μL) and two multiplex approaches (singleplex or two‐plex). Components for each 5 μL PCR1 reaction were 2.5 μL Qiagen Multiplex PCR Plus Master Mix, 1.75 μL extracted DNA, 0.5 μL RNase‐free water, and 0.25 μL 2 μM primers (combined equimolarly), while components for each 10 μL PCR1 reaction were exactly double those for 5 μL reactions. Singleplex reactions were run with all primers that passed the panel refinement step in a single pool, whereas two‐plex reactions were run as two separate PCR1 reactions, each with half of the candidate primers, and then pooled prior to proceeding with PCR2. All PCR1 reactions were run per manufacturer recommendations with an initial activation step of 95°C for 5 min, 35 cycles consisting of a 30‐sec denaturation step at 95°C, a 90‐sec annealing step at 60°C, and a 90‐sec extension step at 72°C, with a 10‐min final extension at 68°C. Following the PCR1 reactions, a 10 μL PCR2 reaction composed of 5.85 μL RNase‐free water, 2 μL NEB OneTaq HotStart Buffer, 0.2 μL 10 mM dNTPs, 0.2 μL of 10 μM N5 index adapter, 0.2 of 10 μM N7 index adapter, 0.05 μL of NEB OneTaq HotStart Polymerase, and 1.5 μL of PCR1 product was then run with an initial activation at 94°C for 2 min followed by 10 cycles consisting of a 30‐sec denaturation step at 94°C, a 60‐sec annealing step at 62°C, and a 60‐sec extension step at 68°C, followed by a 5‐min final extension at 68°C.

Following the PCR steps, we pooled PCR2 products on a per‐plate basis to create a separate pool for each plate. We then performed double‐ended magnetic size selection on a 200 μL aliquot of each pool using Ampure XP beads (Beckman‐Coulter) to remove large fragments using a 0.625× bead: sample ratio, followed by removal of small fragments using a 0.85× ratio and final elution of the size‐selected DNA using 50 μL of Tris–HCl buffer. The DNA concentration of each size‐selected pool was then quantified using fluorometric methods (Promega QuantiFluor ONE dsDNA) and the size distribution was checked using a Aligent 2100 Bioanalyzer. Each plate pool was then combined equimolarly based on quantification results and a second single‐ended size selection step was performed on the ultimate pool using a 0.85× ratio to remove small fragments. This size‐selected pool was then sent to Novogene (Sacramento, CA, USA) for PE150 sequencing on a full Illumina HiSeq X lane nominally yielding 110 Gb of output for a target coverage of 3000× with a 5% PhiX spike‐in.

### 2.4 Data analysis

We demultiplexed loci and called individual genotypes using a python script (amplicon.py, https://bitbucket.org/cornell_bioinformatics/amplicon/src/master/) that has been employed for other microsatellite genotyping projects (Rueger et al., 2021) and itself represents a refinement of the earlier Perl script used by similar studies (D'Aloia et al., 2013; Karn et al., 2021; Marcy‐Quay et al., 2020). In brief, the script separates paired reads by locus using *Cutadapt* (Martin, 2011) and then merges each pair using *BBMerge* (Bushnell et al., 2017). Identical reads are then counted and collapsed, with the ratio of resulting counts used to call haplotypes for each locus and individual. Called genotypes were then processed in *R* (R Core Team, 2021) using the *tidyverse* package for data manipulation and plotting (Wickham et al., 2019). Presence of null alleles and deviations from Hardy–Weinberg equilibrium were assessed using the package *PopGenReport* (Adamack & Gruber, 2014). Population differentiation was analyzed by both calculating pairwise *F*_ST_ using the *hierfstat* package (Goudet, 2005) and performing discriminant analysis of principle components (DAPC; Jombart et al., 2010) using the *adegenet* package (Jombart, 2008) with individuals missing genotyped at more than 10% of loci removed and the remaining missing data imputed as the mean value for that locus. Differentiation was further tested using a second set of known‐origin Champlain and Seneca fish (n = 33 and n = 51, respectively) genotyped using the same panel and methods described in this paper and excluded from DAPC estimation. Kinship inference used the *CKMRsim* package (Anderson, 2020), with allele frequencies calculated using the Seneca and Champlain‐strain hatchery samples in combination with a set of 390 wild‐caught fish from Lake Champlain.

## 3 RESULTS

A total of 186 individuals were amplified using four separate treatments and the resulting 744 unique samples were sequenced. Sequencing yielded 127.0 Gb of raw data corresponding to 853,911,796 reads with an average of 1,138,941 reads per sample (range 24,618–5,694,448). Read counts for sample‐locus combinations were also highly variable with a mean read count of 611 (SD: 5907). A total of 732 samples were able to be genotyped with a further 12 removed because haplotypes could not be called for the majority of loci due to low read depths (fewer than 10 reads per locus). Overall observed heterozygosity (H_O_) was 0.78 while expected heterozygosity (H_S_) was 0.80, with population‐specific metrics showing similar patterns (Table 1).

**TABLE 1:** Marker performance by lake trout population.

Population	Observed heterozygosity (H_O_)	Expected heterozygosity (H_S_)	Average number of alleles	Average allelic richness
Champlain	0.78	0.80	8.09	7.12
Clearwater	0.79	0.78	9.81	6.27
First Bisby	0.76	0.77	7.60	6.60
Keuka	0.78	0.80	9.44	7.27
Klondike	0.79	0.82	8.24	10.00
Parry Sound	0.78	0.81	7.73	8.11
Seneca	0.78	0.80	10.09	6.30
Skaneateles	0.76	0.81	8.10	8.91

*Note*: Allelic richness values were standardized to *N* = 13, the minimum within‐group sample size.

**FIGURE 2:**
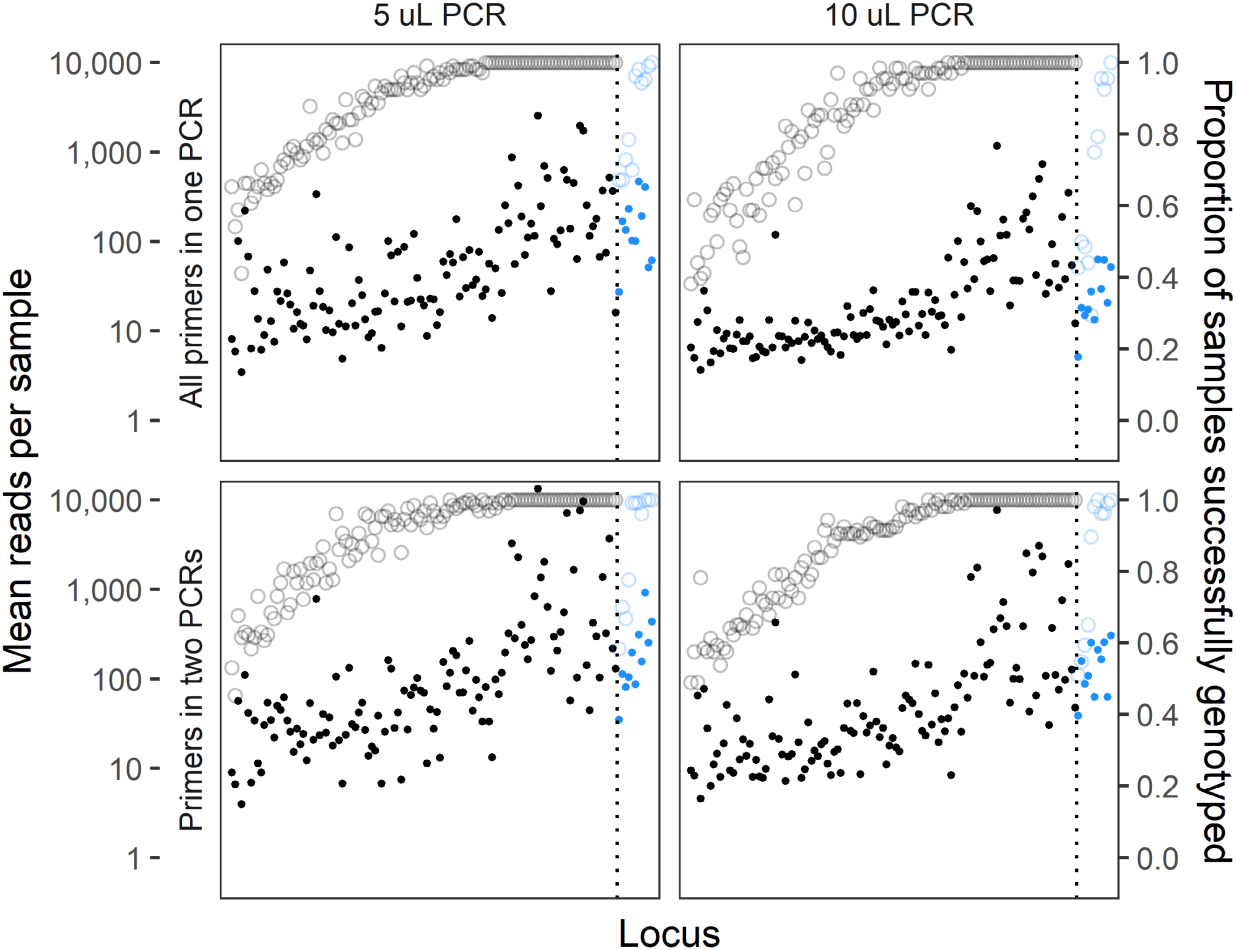
Mean number of sequence reads (filled circles, left axis) and proportion of samples successfully genotyped (open circles, right axis) for each locus by number of PCR reactions (rows) and reaction volume (columns). De novo markers are depicted in black while previously‐published markers are colored blue, with a vertical dotted line separating the two groups. Loci are ordered by the total number of reads among all treatments.

**FIGURE 3:**
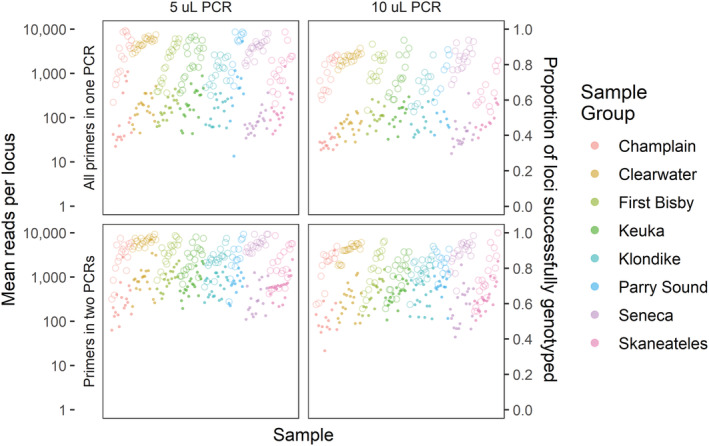
Mean number of sequence reads (filled circles, left axis) and proportion of samples successfully genotyped (open circles, right axis) for each sample by number of PCR reactions (rows) and reaction volume (columns). Samples are grouped and colored by population and ordered by the total number of reads among all treatments.

Distributions of reads retained for genotyping were broadly similar among amplification treatment groups, with loci performing similarly in each group in terms of both total reads and proportion of samples successfully genotyped (Figure 2). In general, 10 μL PCR1 reactions yielded fewer reads than 5 μL reactions, and reactions with primers split into two multiplexes had slightly more successfully genotyped loci. On a per‐sample basis, treatments again showed similar patterns, although the highest read counts were in the two‐multiplex treatment with the single‐multiplex treatments having noticeably lower mean read numbers (Figure 3). Ultimately, however, all treatments produced similar proportions of successfully genotyped loci and samples, suggesting that neither PCR1 volume nor multiplex number strongly influenced the protocol's success at the targeted coverage level.

**FIGURE 4:**
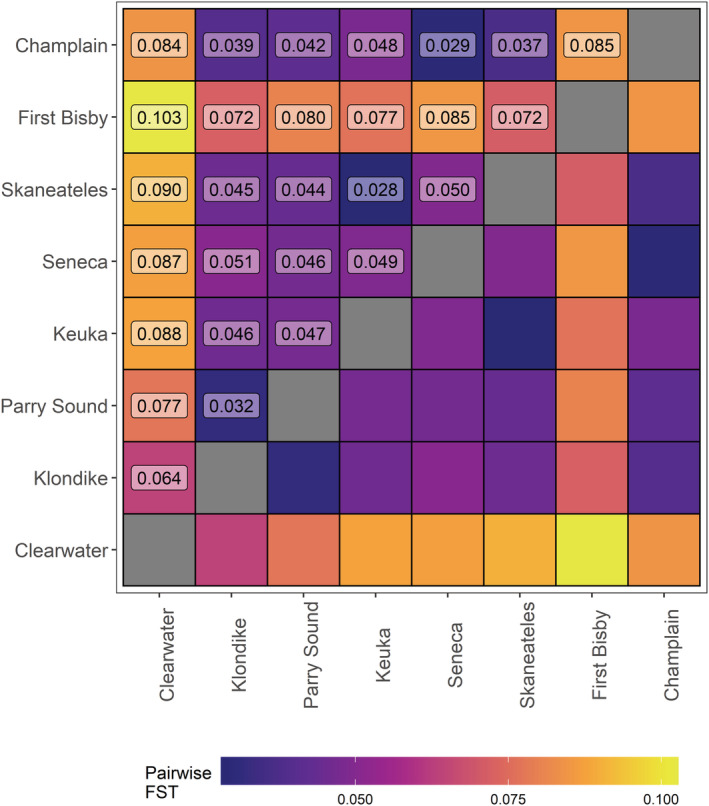
Pairwise *F*_ST_ values for each combination of known‐origin populations in the dataset calculated for the single multiplex, 5 μL reaction treatment group. Populations are ordered by longitude from west to east. The exact value for each combination is provided in the upper triangle of the plot.

Structuring of populations was evident based on pairwise *F*_ST_ results, with the highest differentiation seen between the Clearwater and First Bisby populations (*F*_ST_ = 0.10; Figure 4). Both of these populations were also strongly differentiated from the other populations, with Clearwater individuals appearing most similar to those from Klondike Reef in Lake Superior (*F*_ST_ = 0.06) and the First Bisby individuals appearing most similar to those from Klondike Reef and Skaneateles Lake (*F*_ST_ = 0.07). Populations from the Finger Lakes were broadly similar to each other (average *F*_ST_ = 0.04) and to the Champlain strain which was most closely related to the Seneca population (*F*_ST_ = 0.03), as expected due to the likely origin of this strain from feral Seneca‐strain fish (Ellrott & Marsden, 2004).

**FIGURE 5:**
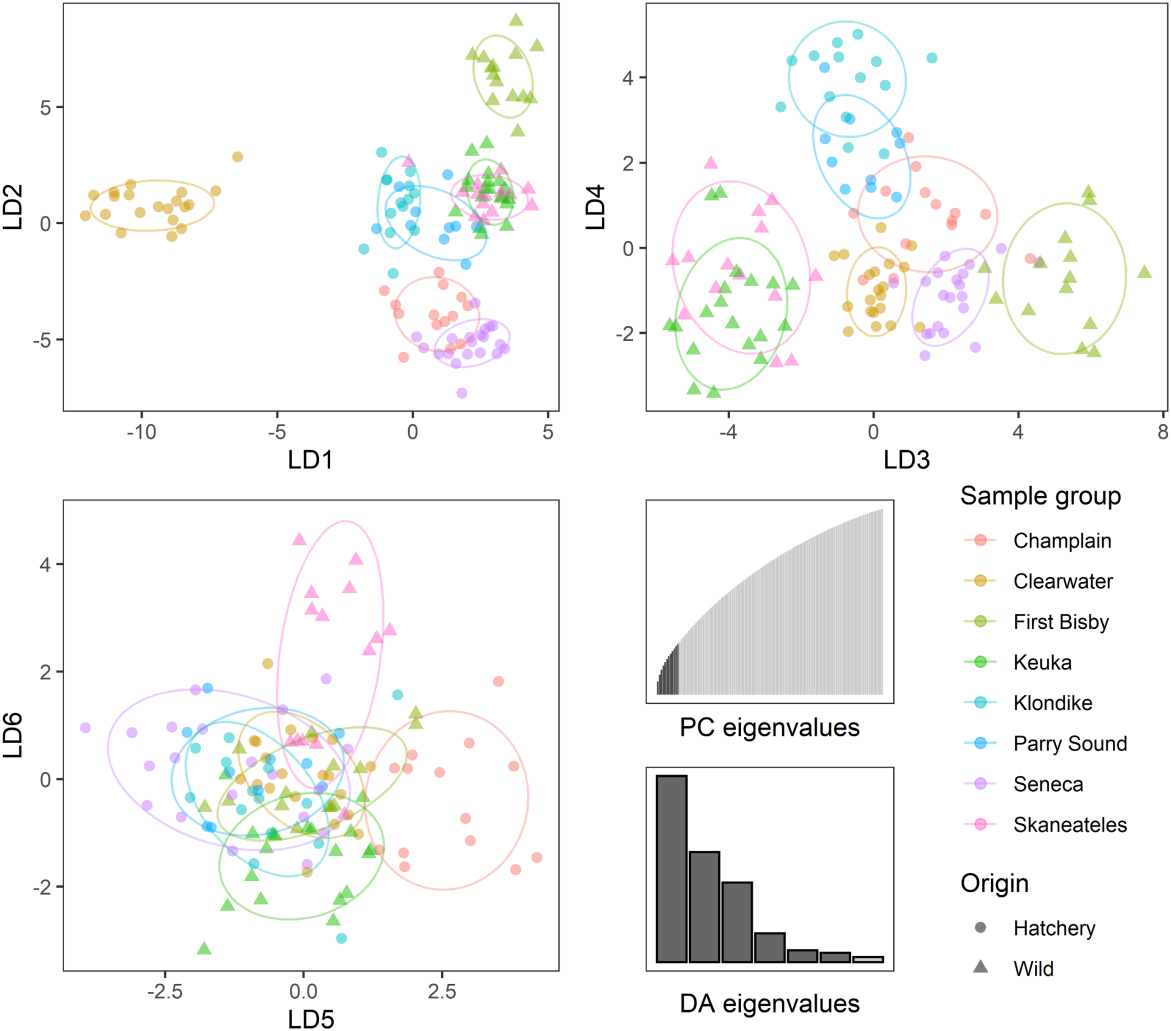
Discriminant principal components analysis for the single multiplex, 5 μL PCR reaction treatment group. Panels show the first six linear discriminant (LD) axes with points representing individual scores and ellipses covering 75% of each cluster, both colored by population. Triangles denote wild‐origin samples while those taken from hatchery fish are depicted by circles. Insets show the distribution of eigenvalues among each discriminant axis (DA) and principal component (PC). Retained DAs and PCs are represented in dark gray.

For the DAPC analysis, we ultimately retained seven principal component axes, corresponding to *K‐1* as recommended by Cullingham et al. (2022). Individuals from each population clustered strongly in the DAPC results, with the first two discriminant axes dominated by the First Bisby and Clearwater outgroups while the third axis and fourth axes separated the remaining populations into three distinct groups: Seneca, Champlain, Klondike and Parry Sound, and Keuka and Skaneateles (Figure 5). The fifth and sixth axes further subdivided these groups, distinguishing Keuka from Skaneateles and Klondike from Parry Sound. Application of the resulting DAPC model to predict assignments for fish in the testing sample set of 84 previously‐excluded Champlain‐ and Seneca‐origin individuals showed high correct assignment rates with 94% of individuals assigned to the correct origin (Table 2).

**TABLE 2:** DAPC‐based assignment performance for Champlain and Seneca validation samples (N = 84).

		Assigned origin	
		Champlain	Seneca
True origin	Champlain	30 (0.94)	2 (0.06)
	Seneca	3 (0.06)	49 (0.94)

**FIGURE 6:**
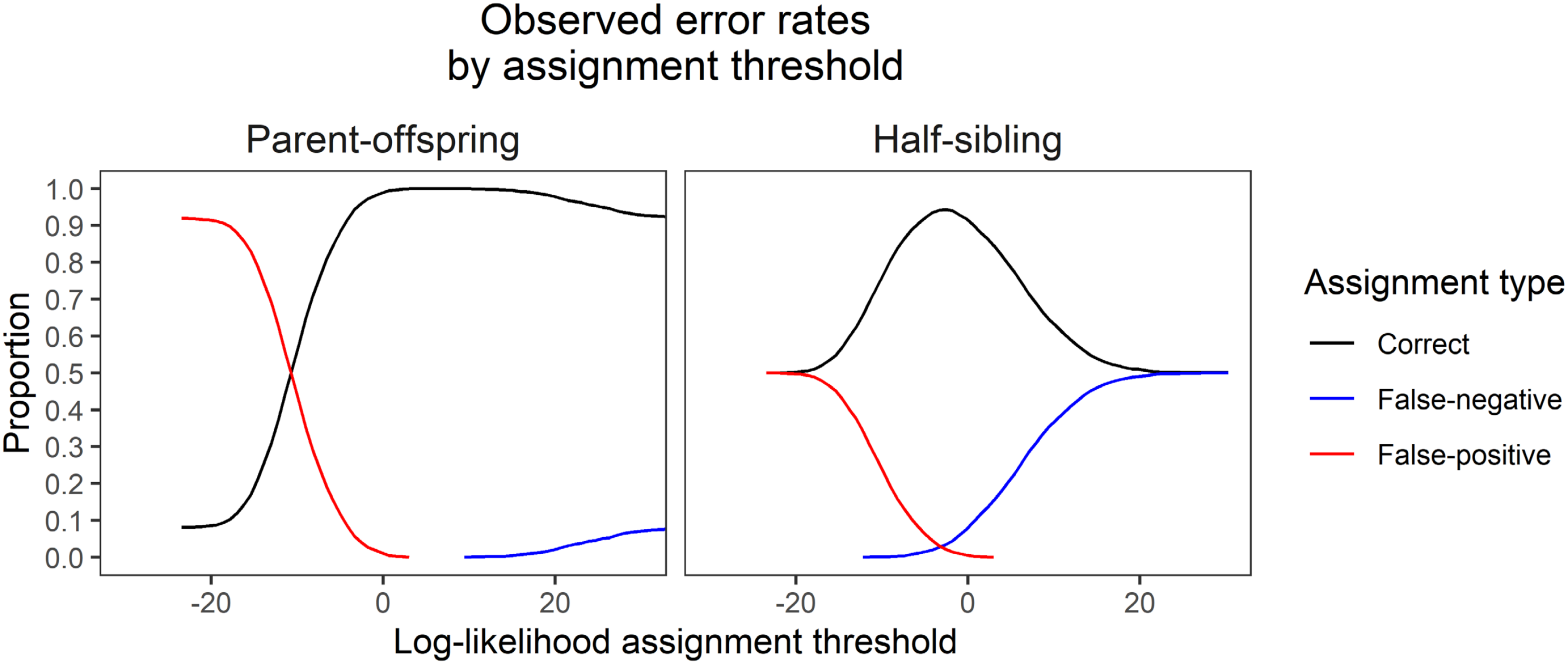
False‐positive and false‐negative rates for parent‐offspring and half‐sibling kinship inference results for the known‐kin validation dataset.

Kinship inference using CKMRsim was highly accurate for parent‐offspring pairs, with log‐likelihood ratios allowing the inference of relationships without any detectable false positives or false negatives (Figure 6). Accuracy for half‐sibling pairs was lower, but still relatively high. The highest overall accuracy was 94.3%, achieved at a false‐positive rate of 2.2%. The lowest detectable false‐positive rate was 0.02%, which corresponded to a false‐negative rate of 15.5% (overall accuracy of 84.5%).

## 4 DISCUSSION

Our results indicate that the marker panel we developed can be used to distinguish among different wild and hatchery‐origin lake trout sources. Both DAPC groupings and pairwise *F*_ST_ values fit expectations, with populations from areas that have historically shared some degree of connection showing less differentiation than those with greater separation by distance or hydrology. Pairwise *F*_ST_ values for populations also analyzed by Larson et al. (2020) were similar, providing further evidence that the new panel provides comparable data to those utilized previously. This included the ability to confidently separate individuals from the Seneca and Champlain strains, a task that was previously difficult using fragment analysis‐based microsatellite (Markham et al., 2022; Salvesen, 2015) due to high proportion of Seneca‐strain individuals present in the feral spawning fish collected to establish the broodstock (Ellrott & Marsden, 2004).

In addition to providing high‐resolution information on population structure, our panel also has the necessary power to accurately infer parent‐offspring and half‐sibling relationships. This capability provides the foundation for kinship‐based population analysis techniques such as CKMR and parentage‐based tagging. The former technique is especially demanding in terms of kinship precision because it requires extremely low false‐positive rates due to the expected number of true positives usually being several orders of magnitude lower than the expected number of true negatives (Bravington, Grewe, & Davies, 2016). Validation with a set of hatchery‐raised known‐origin individuals showed that the panel was able to meet this criterion with a 0% apparent false‐positive rate for parent‐offspring pairs without producing any false negatives. Apparent error rates for known‐origin half‐siblings were higher, but still usable for CKMR given a priori knowledge of expected error rates. These rates are similar to those from other studies. For example, ongoing CKMR‐based southern bluefin tuna management uses a set of approximately 2000 SNP loci yielding an estimated false‐negative rate between 10.5% (Bravington et al., 2017) and 25% (Farley et al., 2021). In any event, if false‐negative rates are well‐characterized they can be allowed for during the population modeling stages of CKMR by increasing the amount of “true” pairs to account for missed detections (Farley et al., 2021). It should also be noted that these rates represent a worst‐case scenario because the crosses were derived from six feral spawning fish caught from a single shoal in Lake Champlain. As previously mentioned, Seneca‐strain and Champlain‐strain fish are highly similar, and analysis with our panel suggests that four out of the six fish should be assigned to the Champlain strain. Thus, the “unrelated” fish in our known‐origin validation dataset are likely far more genetically similar than would be expected for a population originating from sustained natural reproduction.

Analysis of pairwise F_ST_ revealed interesting patterns likely to reflect zoogeography and introgression due to historical stocking, or lack thereof. For example, isolation by distance was clearly apparent in the strong differentiation among fish from Clearwater Lake, a pure strain endemic to Manitoba, Canada, and all other populations. Likewise, the lowest pairwise F_ST_ values for Clearwater Lake were for comparisons with the two geographically closest sampled populations, Klondike and Parry Sound, which are both derived from the upper Great Lakes. We observed similarly high *F*_ST_ values among the individuals from First Bisby Lake and all other populations. This suggests that First Bisby fish are indeed native as listed by Thill et al. (2014), despite records indicating that the chain of lakes was stocked at least once (and possibly more times) with fish taken from Lake Huron (Mather, 1886). Samples from First Bisby showed the least differentiation from Skaneateles Lake individuals, consistent with geographic separation of native strains, as First Bisby Lake and Skaneateles Lake are only approximately 150 km apart and both are part of the larger Lake Ontario watershed. Interestingly, samples from Seneca Lake were more differentiated from both Skaneateles and Keuka than either were from each other, despite the much larger Seneca Lake lying between the two and records indicating that Keuka and likely Skaneateles were historically stocked with Seneca‐origin fish (Fitzsimons et al., 2005). The reasons for this apparent discontinuity are unclear, but Seneca Lake fish appear to differ substantially from those in the Adirondacks and other Finger Lakes in both appearance and spawning habits (Royce, 1951; Sly & Widmer, 1984); Seneca Lake fish may represent a regional outlier with behavioral differences that limited their ability to introgress into other Finger Lakes strains (i.e., prezygotic barriers).

Our results indicate that two potential protocol modifications, incorporation of all primers into a single PCR reaction and a reduced reaction volume of 5 μL, can be adopted with little effect on overall per‐sample success rates. These changes further reduce the cost of what is already a highly economical and efficient approach for high‐throughput genotyping. We estimate that our protocol costs approximately $3.00 USD per sample and requires an average of 3 minutes of hands‐on work. Of this, DNA extraction accounts for $0.05 and 75 seconds per sample, with the bulk of labor being the sectioning of tissue samples. The initial amplification reaction with locus‐specific primers requires an additional $0.50 and 20 seconds per sample, representing a 75% decrease in cost and 50% decrease in labor due to the two protocol modifications. Adapter ligation adds a further $0.45 and 40 seconds per sample and requires the most skill due to the high potential for unrecoverable errors if contamination occurs between wells. Finally, sequencing represents the bulk of the cost with a target coverage of 3000× requiring approximately 0.13 Gb of sequence per sample. The wide variety of sequencing options and quickly‐changing market make this component the most uncertain. In our study, we made use of the HiSeq X platform with list prices of approximately $15/Gb and a capacity of roughly 768 samples (eight plates) per full lane run resulting in a per‐sample cost of roughly $2.00. Projects involving more samples may be able to make use of higher‐capacity sequencers that are more economical on a per‐sample basis, such as the NovaSeq 6000 S4 lanes. Likewise, smaller projects will necessitate less efficient sequencing options and therefore incur higher per‐sample costs unless they can be combined with other projects (i.e., partial‐lane sequencing).

The lack of any per‐sample normalization steps in our protocol is a deliberate choice, trading the cost and time involved in quantification and pooling for a higher target sequencing coverage. While per‐sample costs for current dsDNA quantification methods vary widely, the majority of methods are more costly than all steps of our protocol combined (Hussing et al., 2018). The one method tested by Hussing et al. (2018) that was less expensive (Nanodrop; $0.50 per‐sample) still required an extra 30 seconds per‐sample for quantification alone, representing at least a 17% increase in preparation time before normalization is taken into account. Likewise, while bead‐based normalization techniques can provide significant time‐ and cost‐savings (Hosomichi et al., 2014), the addition of these techniques would still represent an outsized proportion of the overall resources needed to complete our protocol. Instead, targeting higher sequencing depth allowed us to call genotypes with a relatively low failure rate (1.6%) despite eschewing normalization steps. Without this normalization and due to differing amplification efficiency between loci, the number of reads for each sample‐locus combination varies substantially (Figures 2 and 3) but the majority of combinations still provide reads for a genotype call. For those samples that did fail, the efficiency of the protocol means that samples can be easily re‐run.

In summary, we have created an efficient amplicon sequencing‐based microsatellite marker panel for the study of lake trout population genetics. The additional resolution afforded using high numbers of microsatellites allowed us to accurately infer kinship and resolve detailed patterns of differentiation among both geographically widespread and adjacent populations. By optimizing our genotyping protocol, we were also able to reduce per‐sample costs to a point that is affordable for a range of study sizes. As the remaining cost is dominated by sequencing expenses, future improvements in technology may provide further cost reductions. Preliminary results suggest that many of the included loci may also amplify for other *Salvelinus* species, with a roughly 75% success rate observed when applied to a set of 20 samples from brook trout (*Savelinus fontinalis*; B. Marcy‐Quay, unpublished data). Ultimately, this panel provides a foundation for future studies into the population dynamics and biogeography of lake trout throughout their current distribution with potential extension to other closely related species.

## AUTHOR CONTRIBUTIONS

**Benjamin Marcy‐Quay:** Conceptualization (lead); data curation (lead); formal analysis (lead); funding acquisition (lead); investigation (lead); methodology (lead); software (lead); validation (lead); visualization (lead); writing – original draft (lead); writing – review and editing (equal). **Chris C.C. Wilson:** Conceptualization (supporting); funding acquisition (supporting); methodology (supporting); writing – review and editing (equal). **Christopher Osborne:** Resources (supporting); writing – review and editing (equal). **Ellen Marsden:** Conceptualization (supporting); funding acquisition (lead); methodology (supporting); project administration (lead); resources (lead); supervision (lead); writing – review and editing (equal).

## Supporting information


Table S1.

## Data Availability

Genetic data and sample metadata are archived and publicly accessible at https://doi.org/10.5061/dryad.msbcc2g38. Primer sequences are provided in the Supporting Information for this article. The genotyping pipeline script is available via the link included in the methods section (https://bitbucket.org/cornell_bioinformatics/amplicon/src/master/).
